# Evaluation of derivative spectra for the selective determination of drugs: quantitation of theophylline with phenobarbital and light-scattering components

**DOI:** 10.1155/S1463924686000160

**Published:** 1986

**Authors:** Peter B. Arnoudse, Harry L. Pardue

**Affiliations:** Department of Chemistry Purdue University West Lafayette Indiana 47907 USA


Journal of Automatic Chemsitry/*Journal of Clinical Laboratory Automation*, Vol. 8, No. 2 (April–June 1986), pp. 75–79

### Evaluation of derivative spectra for the selective determination of drugs: quantitation of theophylline with phenobarbital and light-scattering components

**Peter B. Arnoudse and Harry L. Pardue***

*Department of Chemistry, Purdue University, West Lafayette, Indiana 47907, USA*

One of the significant developments in analytical instrumentation in recent years has involved the adaptation of imaging detectors for quantitative spectroscopy [1 and 2]. These detectors make it possible to scan optical spectra more rapidly than is possible with conventional instrumentation. Commercially available diode-array based instrumentation makes it possible to scan the spectrum from 200 to 800 nm with repetition rates of one scan/s. The instrumentation includes computer software to compute first- and second-derivative spectra and to resolve multicomponent mixtures with multiwavelength curve-fitting methods [3]. This instrumentation offers measurement and data-processing options that are difficult to implement with conventional spectrophotometers.

The purpose of this work was to evaluate and compare some options offered by this new generation of spectrophotometers for the quantitation of drugs. Specifically, a goal was to compare the relative merits of zeroth-, first-, and second-derivative spectra used in single-wavelength and multiwavelength modes. To date, most applications of derivative spectroscopy have involved single-wavelength measurements [4 and 5]. Such approaches under-utilize the information content of derivative spectra, just as they do for absorption spectra, and they are also susceptible to larger random errors that result from the increased noise imposed by the derivative process. Multiwavelength data-processing methods applied to derivative spectra should improve both the selectivity and random errors associated with derivative spectroscopy.

The example system chosen for this study involves the quantitation of theophylline in the presence of phenobarbital. Because theophylline is sometimes administered with phenobarbital, this example has practical significance in clinical chemistry. An earlier paper described an extraction procedure to isolate these drugs from other serum components and a two-wavelength measurement method to quantify theophylline in the presence of phenobarbital and background (blank) absorption signals [6]. The primary focus in this work was on the evaluation of multiwavelength derivative spectra to quantify theophylline in such media without the pH-adjustment step required in the previous procedure [6]. A controlled background spectrum was simulated by adding known amounts of a light-scattering component (kaolin) to samples.

* Correspondence to Professor Pardue.

### Experimental

### *Instrumentation*

All experiments were performed with a diode-array-based Hewlett-Packard 8450A UV/VIS spectrophotometer (Hewlett-Packard Company, Palo Alto, California, USA). The operating software for the spectrophotometer contains programs to compute the first and second derivatives of absorbance, with respect to wavelength, and contains single- and multi-component concentration routines.

### *Reagents*

Primary stock solutions containing 1000 mg/l theophylline (Sigma Chemical Company, St. Louis, Missouri, USA) and phenobarbital (U.S.P., Mallinckrodt, St. Louis, Missouri, USA) were prepared in methanol and stored at −18° C. Secondary stock solutions containing 50 mg/l theophylline and phenobarbital were prepared daily by diluting the primary stock solutions with 0·1 M NaOH. A stock kaolin suspension, 0·125 mg/l, was prepared by suspending kaolin (particle size 0·1–0·4 μm, Sigma Chemical Company) in 0·1 M NaOH and sonicating the suspension for 15 min to aid dispersion. Single- and multiple-component standards were prepared daily by diluting these stock solutions with 0·1 M NaOH to concentrations of 0 to 25 mg/l theophylline, 0 to 25 mg/l phenobarbital and 0 to 50 mg/l kaolin.

### *Procedures*

All spectra were monitored over a 200 to 80 nm wavelength range with a 10 s integration period and stored on cassette-tape for subsequent processing. First and second derivatives of absorbance, with respect to wavelength, were computed in the spectrophotometer by a routine using differences between data points. The concentrations of theophylline and phenobarbital were calculated using single- and multiple-component concentration routines included as part of the software in the spectrophotometer. The single-component concentration routine used assumes a linear calibration curve. The multi-component concentration routine calculates concentrations that, when combined with spectra for pure components, give a best (least-squares) fit to the experimental data over the wavelength range of interest.

For the single-component routine, the wavelengths were selected to maximize the response of the component of interest relative to the response of the other components, i.e. wavelengths were selected for which the component of interest had a maximum absolute response or for which the response of the other components approached zero. For theophylline, the wavelengths used for single-wavelength quantitation were 275 nm for absorbance data, 287 nm for first derivative data, and 296 nm for second derivative data. For the multi-component routine, the wavelength ranges were selected to include the unique spectral characteristics of both theophylline and phenobarbital. The wavelength ranges used were 235 to 300 nm for absorbance data, 235 to 305 nm for first-derivative data, and 240 to 310 nm for second-derivative data.

### Results and discussion

Figures 1(*a*)–1(*c*) show the spectra of the three components included in this study. Although it is possible to shift the absorption band for phenobarbital to shorter wavelengths by reducing the pH [6], our primary interest here was an evaluation of the relative merits of the zeroth-, first-, and second-derivative spectra for the quantitation of components with overlapping spectra without the inclusion of chemical steps to alter the spectra.

Comparing the plots, it is apparent that both the first- and second-derivative spectra have regions in which the signal for one of the drugs is emphasized relative to the other components, while there is no region in the absorption spectrum in which either drug can be quantified without interference from the other two components. The goal here was to evaluate different approaches that can be used to exploit the enhanced selectivity of the derivative spectra. Primary emphasis was on quantitation of theophylline [6].

### *Single-wavelength results*

To date, most applications of derivative spectroscopy have involved measurements at single wavelengths at which the derivative spectra are responsive only to the component of interest. Spectra in figures 1(*b*) and 1(*c*) suggest that measurements at 287 nm (figure 1[*b*]) and 296 nm (figure 1[*c*]) should be appropriate for quantitation of theophylline with first- and second-derivative spectra, respectively.

Figure 2 presents calibration data for theophylline quantified in the presence of fixed amounts of phenobarbital and the clay suspension with absorbance, first- and second-derivative spectra. Although all plots are linear, it

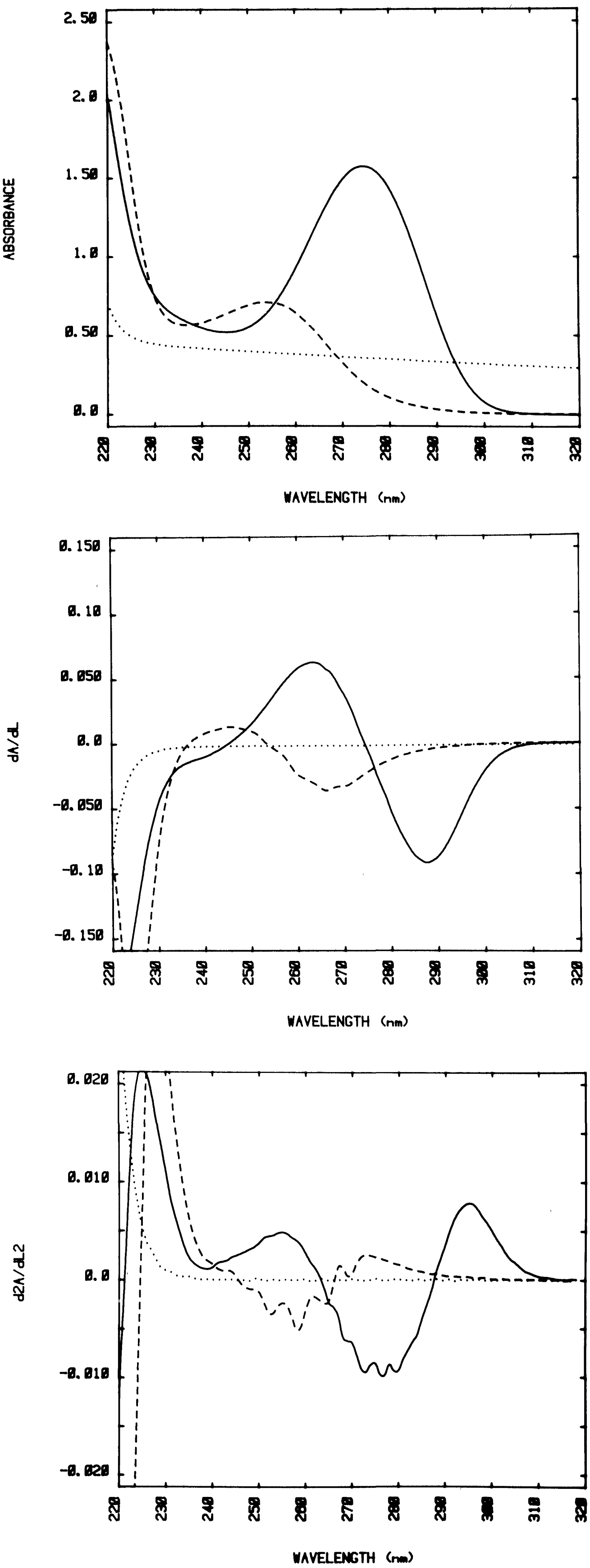

*Figure 1. Spectral data for phenobarbital, theophylline, and kaolin. All frames: — theophylline (25·0 mg/l), - - - phenobarbital (25·0 mg/l), . . . kaolin (50·0 mg/l). (a) Absorbance, (b) First derivative, (c) Second derivative.*

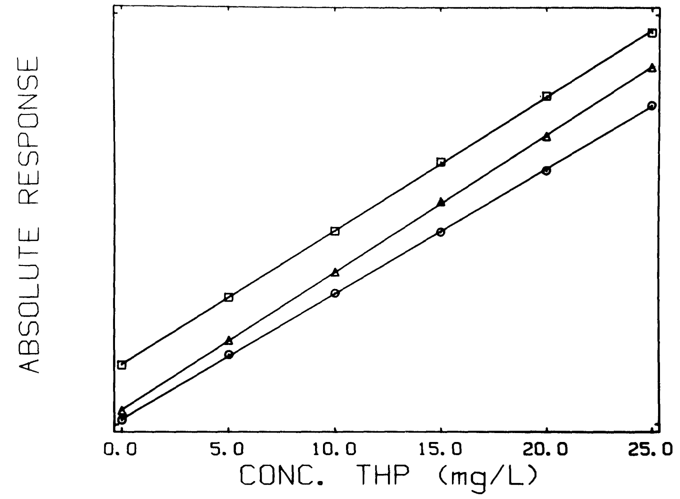

*Figure 2. Response versus concentration for theophylline. Phenobarbital (12·5 mg/l) + kaolin (25·0 mg/l) Absorbance (□, 275 nm, ordinate 0 to 1·95); first derivative (△, 287 nm, ordinate 0·00 to 0·11); second derivative (○, 296 nm, ordinate 0·000 to 0·010).*

is noted that the intercepts decrease as the order of the derivative increases. These intercepts reflect the extent to which the fixed concentrations of the other two components interfere with the quantitation of theophylline. The second-derivative data exhibit the smallest degree of interference. In table 1, the first three rows of data present least-squares statistics for the computed ($y$) · versus prepared ($x$) concentrations of theophylline. Although the three data modes yield similar slopes (near unity), standard errors of estimate, and correlation coefficients, intercepts decrease significantly as the order of the derivative increases, again reflecting the improved ability of higher order derivative spectra to reduce effects of potential interferents.

Additional experiments were performed in which concentrations of all three components were varied. Results for first- and second-derivative data are illustrated graphically in figure 3, and for all data modes with least-squares

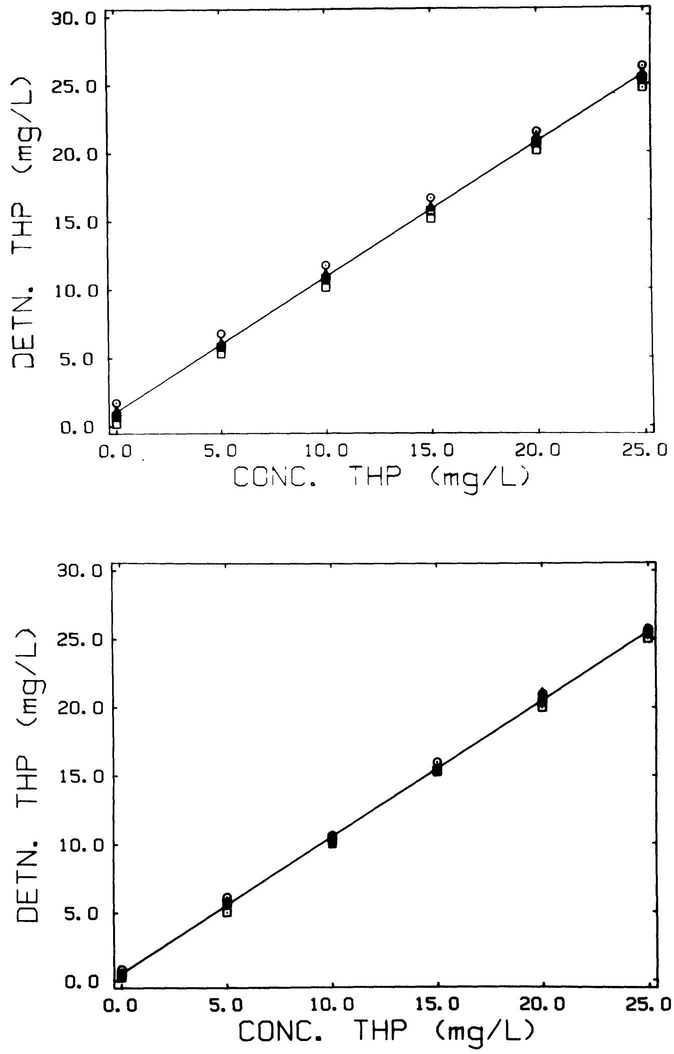

*Figure 3. First- and second-derivative responses for theophylline in the presence of variable amounts of phenobarbital and kaoline. Phenobarbital, kaolin (mg/l): □ (0·0, 25·0), △ (12·5, 25·0), ◇ (25·0, 25·0), □ (12·5, 0·0), △ (12·5, 50·0). (a) first derivative; (b) second derivative.*

*Table 1. Least-squares statistics for quantitation of theophylline.*

| Derivative order | Wavelength (nm) | Slope ± s.d. | Intercept ± s.d. (mg/l) | Standard error estimation (mg/l) | Correlation coefficient |
|---|---|---|---|---|---|
| | | Single wavelength-fixed concentration of interferent[a] | | | |
| Zeroth | 275 | 0·969 ± 0·006 | 4·21 ± 0·09 | 0·12 | 0·9999 |
| First | 287 | 0·988 ± 0·006 | 1·05 ± 0·08 | 0·12 | 0·9999 |
| Second | 296 | 1·001 ± 0·005 | 0·58 ± 0·07 | 0·10 | 0·9999 |
| | | Single wavelength-variable concentration of itnerferent[b] | | | |
| Zeroth | 275 | 0·96 ± 0·04 | 4·24 ± 0·62 | 1·9 | 0·9754 |
| First | 287 | 0·98 ± 0·01 | 1·05 ± 0·17 | 0·52 | 0·9982 |
| Second | 296 | 1·000 ± 0·007 | 0·52 ± 0·10 | 0·32 | 0·9994 |
| | | Multiwavelength-variable concentration of interferent[b] | | | |
| Zeroth | 235–300 | 1·08 ± 0·05 | 2·1 ± 0·8 | 2·6 | 0·966 |
| First | 235–305 | 0·986 ± 0·003 | 0·19 ± 0·05 | 0·15 | 0·9998 |
| Second | 240–310 | 0·982 ± 0·002 | 0·04 ± 0·03 | 0·09 | 0·9999 |

| | No. of samples | Concentrations (mg/l) Theophylline | Phenobarbital | Kaolin |
|---|---|---|---|---|
| a | 6 | 0–25 | 12·5 | 25 |
| b | 30 | 0–25 | 0–25 | 0–50 |

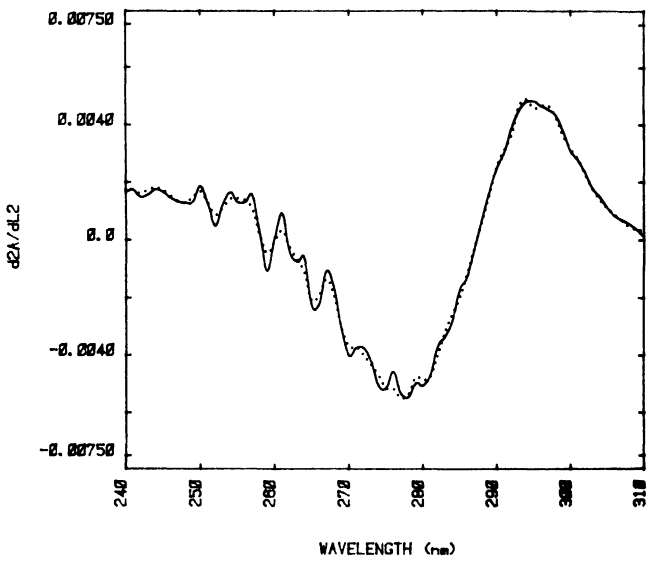

*Figure 4. Second derivative spectrum for mixture of theophylline (15·0 mg/l), phenobarbital (12·5 mg/l), and kaolin (25·0 mg/l). — Experimental, · · · fitted.*

statistics in the second set of three rows in table 1. The decrease in scatter about the least-squares line in figure 3(*b*) relative to 3(*a*) reflects the improved ability of the second-derivative data to compensate for variable amounts of interferences. A plot for absorbance data would be similar to these, except that the scatter about the line would be much larger than for either data set in figure 3. These differences are reflected quantitatively in the standard errors of estimate of the least-squares data in table 1. Comparing statistics for variable and fixed amounts of interferents, it is noted that the standard errors of estimate differ by factors of about 16 for absorbance data, about four for first-derivative data, and about three for second-derivative data.

With carefully selected wavelengths, it is possible to resolve theophylline (or phenobarbital) in the presence of the other two components using either first- or second-derivative data, with second derivative data yielding the better selectivity. However, single wavelengths must be selected very carefully. For example, first derivative data at 253 nm yielded excellent linearity for theophylline, but a comparison of computed versus prepared concentration yielded a slope of 0·91 for both data sets described above, rather than the expected value of unity. Also, single-wavelength derivative data are susceptible to increased noise relative to absorbance data. For these reasons, multiwavelength data-processing methods were also evaluated.

### *Multiwavelength results*

The multiwavelength data-processing methods originally developed for absorbance data [3] are also applicable to first- and second-derivative data. Expected advantages are improved signal averaging relative to single-wavelength results, improved selectivity in regions where spectra for potential interferences have little curvature (second derivative) or low slopes (first derivative) relative to the component(s) of interest, and the ability to quantify multiple components in mixtures.

In this study, spectra for all the components were available, and multiwavelength methods could be used to compensate for the effects of the scattering component on absorbance data. However, in real samples, such as extracted sera, the spectrum of the scattering component would not be available, and attention was focused on the relative abilities of derivative spectra to nullify effects of scattering components. To do this, multiwavelength derivative data for the mixture discussed in the previous section were processed without assuming any prior knowledge of the spectra of the scattering component; the three-component mixtures were treated as two-component mixtures by ignoring contributions from the scattered light.

Figure 4 shows a typical second-derivative spectrum for a three-component sample with a fitted spectrum based on a two component model. The smoothing effect of the curve-fitting process is apparent at shorter wavelengths at which there is increased noise in the derivative spectrum. Except for this smoothing effect, there is good agreement between experimental and fitted data.

Least-squares statistics for theophylline in samples with variable amounts of the other components are summarized in the last three rows of table 1. Results obtained with absorbance data reflect the expected interference from the scattering component which is not taken into account in the calculation. Results for first- and second-derivative data exhibit a slightly smaller deviation of the slopes from the expected values of unity and all other statistics including the intercepts, standards deviations of interecepts, standard errors of estimate, and correlation coefficients are also improved. Either first- or second-derivative spectra used with the multiwavelength data-processing method yield excellent results for theophylline in these mixtures. The authors' experience indicates that this measurement/data-processing approach is more than adequate to satisfy the requirements of the previously described extraction procedure [6]. It is also possible to quantify phenobarbital in the extract, although results are less reliable than those for theophylline. This decrease in reliability is believed to be a result of the pH dependence exhibited by the ultra-violet absorption (i.e. wavelength, intensity and stability) of phenobarbital in alkaline solution [7], rather than a limitation of the method.

### Acknowledgement

This work was supported by a grant from the National Institute of Health. Contract #GM 13326–18.

### References

1. Talmi, Y., *Analytical Chemistry*, **47** (1975), 658A.
2. Pardue, H. L., In *Topics in Automatic Chemical Analysis*, J. K.

Forman and P. B. Stockwell (Eds) (Ellis Horwood Publishers, Chichester, England (1979), 163.
3. STERNBERG, J. S., STELLO, H. S., and SCHWENDEMAN, R. H., *Analytical Chemistry*, **32** (1960), 84.
4. HAGER, R. N., *Analytical Chemistry*, **45** (1973), 1131A.
5. O'HAVER, T. C., *Analytical Chemistry*, **51** (1979), 91A.
6. JATLOW, P., *Clinical Chemistry*, **21** (1975), 1518.
7. HAMILTON, H. E., and WALLACE, J. E., In *Antiepiletic Drugs: Quantitative Analysis and Interpretation*, C. E. Pippenger, J. K. Penry, and H. Kutt (Eds) (Raven Press, New York) 1978, 175.

### ANATECH 86

*To be held in Noordwijkerkout, The Netherlands from 22 to 24 April 1986.*

*Scope*

- The importance of analytical techniques for the control of industrial processes is steadily increasing.
- The development of process analysers had taken place to a great extent outside the analytical laboratory. This has led to a distinct gap between experts in the field of process analysis and analytical chemists. Bridging this gulf is one of the main objectives of this conference.
- Analytical chemists have to recognize the need for instruments for process control and the special demands imposed on them, and process and system engineers need to be informed about analytical techniques that may be or may become of interest for process control purposes.
- In addition to techniques already generally accepted, such as process chromatography, IR-analysers, conductometry etc., it is intended to emphasize the possible role of chemical type analysers.
- The symposium is aimed at an interdisciplinary audience of industrial and academic analytical chemists and those involved in process control and process analysis.

*Scientific programme*

The scientific programme will consist of invited plenary lectures, keynote lectures and submitted research papers. Invited lectures are:

A review of on-line analysers (J. R. P. Clarke, UK)
Monitoring problems and their relation to the teaching of analytical chemistry (E. Pungor, Hungary)
Philosophy underlying the choice and installation of on-stream analysers with emphasis on IR-instruments (M. Pochon, Switzerland)
Automated analysis in research: a link between laboratory and process analysis with emphasis on chromatography (H. Hachenberg, FR Germany)
The contribution of quality aspects to process control (J. E. Rijnsdorp, The Netherlands)
The capital notion of sampling correctness (P. Gy, France)
The estimation of the appropriate sampling frequency (F. W. Pijpers, The Netherlands)
References in development, application and marketing of industrial process analysers (M. Jola, Switzerland)
The evaluation of analysis systems (P. van der Louw, The Netherlands)
Experiences with process analysers in the chloralkaline industry (H. van den Dolder, The Netherlands)
Process analysers based on chemical reactions (G. Johansson, Sweden)
Future developments in process analytical chemistry (T. Hirschfeld, Livermore, USA).

*Details from Professor Dr van der Linden, Laboratory for Chemical Analysis, Department of Chemical Technology, Twente University of Technology, PO Box 217, NL 7500 AE Enschede, The Netherlands.*

